# Excellent accuracy of magnetic resonance imaging for diagnosis of discoid meniscus tears: A systematic review and meta‐analysis

**DOI:** 10.1002/jeo2.12051

**Published:** 2024-06-19

**Authors:** Shayan Amiri, Alireza Mirahmadi, Ava Parvandi, Mana Zaker Moshfegh, Seyedeh Pardis Hashemi Abatari, Mehrdad Farrokhi, Seyyed Mehdi Hoseini, Seyed Morteza Kazemi, Kaveh Momenzadeh, Jim S. Wu, Ara Nazarian

**Affiliations:** ^1^ Shohadaye Haftom‐e‐Tir Hospital, Department of Orthopedic, School of Medicine Iran University of Medical Sciences Tehran Iran; ^2^ Bone, Joint and Related Tissue Research Center, Akhtar Orthopedic Hospital Shahid Beheshti University of Medical Sciences Tehran Iran; ^3^ Student Research Committee, Department of Epidemiology, School of Public Health and Safety Shahid Beheshti University of Medical Sciences Tehran Iran; ^4^ Department of Orthopaedic Surgery, Boston Children's Hospital and Harvard Medical School, Boston the Musculoskeletal Translational Innovation Initiative Boston Massachusetts USA; ^5^ Department of Radiology, Beth Israel Deaconess Medical Center Harvard Medical School Boston Massachusetts USA

**Keywords:** diagnostic imaging, discoid meniscus, discoid meniscus tear, magnetic resonance imaging, meta‐analysis, sensitivity and specificity

## Abstract

**Purpose:**

The discoid meniscus (DM) is distinguished by its thickened, disc‐shaped formation, which extends over the tibial plateau. The likelihood of developing osteoarthritis escalates if a DM tear remains undiagnosed and untreated. While DM tears can be diagnosed through arthroscopy, the high cost, invasive nature and limited availability of this procedure highlight the need for a better diagnostic modality. This study aims to determine the accuracy of magnetic resonance imaging (MRI) in diagnosing DM tears.

**Methods:**

A systematic review was conducted to gather articles with at least 10 cases on the comparison of MRI and arthroscopy as the gold standard for DM tear diagnosis. Stata and MetaDisc were used to conduct the statistical analysis. The quality of the included studies was evaluated using the Quality Assessment of Diagnostic Accuracy Studies‐2 tool.

**Results:**

Five diagnostic performance studies, derived from four original research papers involving 305 patients, were evaluated. Based on the pooled data, the sensitivity, specificity, diagnostic odds ratio, positive limit of detection and negative limit of detection were found to be 0.87 (95% confidence interval [CI], 0.82–0.91) and 0.84 (95% CI, 0.75–0.90), 32.88 (95% CI, 5.81–186.02), 5.22 (95% CI, 1.71–15.92) and 0.18 (95% CI, 0.09–0.38), respectively. A hierarchical summary receiver operating characteristic curve with an area under the curve of 0.92 was generated.

**Conclusion:**

This meta‐analysis demonstrates that MRI has excellent sensitivity and specificity for diagnosing DM tears. Despite its lower accuracy compared to arthroscopy, MRI can be used in symptomatic patients as a viable alternative to arthroscopy due to its inherent advantages.

**Level of Evidence:**

Level IV.

**Abbreviations**
DM: discoid meniscus
DOR: diagnostic odds ratio
LOE: level of evidence
MRI: magnetic resonance imaging
NLR: negative likelihood ratio
PLR: positive likelihood ratio
PRISMA‐DTA: Preferred Reporting Items for Systematic Reviews and Meta‐Analyses for Diagnostic Test Accuracy
PROSPERO: The International Prospective Register of Systematic Reviews
QUADAS‐2: Quality Assessment of Diagnostic Accuracy Studies‐2

## INTRODUCTION

A functioning meniscus has a profound effect on both the kinematics of the knee as well as the long‐term maintenance of the chondral surfaces [18, 22, 26]. The lateral meniscus has a greater number of variants compared to the medial meniscus, with the discoid meniscus (DM) being the most prevalent type among these variants [33]. DM is characterized by a thickened, disc‐shaped structure that covers a larger area of the tibial plateau [38]. Approximately 0.4%–20% of the population have DM [52]. Following an unstable meniscal tear, typically occurring between ages 8 and 9, patients usually experience no symptoms in childhood except for snap‐knee syndrome [8, 14, 39, 51]. Surgical interventions such as partial meniscectomy, saucerization and direct repair may be necessary when a symptomatic DM is present [15, 28]. However, surgical intervention is unnecessary when discoid menisci are discovered incidentally during arthroscopic knee surgery [3]. It has been shown that enlargement and thickening of the DM caused by atypical processes and abnormal peripheral attachments can result in premature tissue degeneration [47].

Mechanical trauma is more likely to occur in DM because of its thickness and low vascularization. However, patients with DM tears might not have a history of traumatic events [47]. The frequency of tears associated with discoid menisci ranges from 38% to 88% [3, 4, 40]. A wide range of physical findings, imaging findings and treatment methods are also known for each type of tear. A detailed description of the inverted type of discoid lateral meniscus (DLM) tear has yet to be provided [20]. There is no evidence that a specific presentation of symptoms indicates a torn DM; however, persistent knee pain, clicking or snapping of the knee and decreased function can suggest DM tears [12]. The possibility of osteoarthritis increases when persistent symptoms are caused by a meniscal tear not detected during initial surgery [36].

While meniscal tears can be diagnosed using magnetic resonance imaging (MRI), their effectiveness remains unclear. According to some authors, there is still uncertainty regarding the routine use of MRI, which can be challenging, and arthroscopy is commonly suggested for both diagnostic and therapeutic purposes. Even though arthroscopy is the gold standard for DM tears [35], its disadvantages, such as high cost, invasive nature and limited availability, increase the need for a better diagnostic option. While MRI is available as a diagnostic option, the accuracy and reliability of the results are yet to be established to evaluate whether MRI may replace or be complementary to arthroscopy. The accuracy of MRI in detecting meniscus lesions has been evaluated for some other conditions, such as ramp lesions [27], but no previous study has evaluated MRI accuracy in detecting DM tears. This study aims to evaluate the sensitivity and specificity of MRI in detecting DM tears. The MRI was expected to help diagnose DM tears with high accuracy.

## METHODS

Following PRISMA‐DTA (Preferred Reporting Items for Systematic Reviews and Meta‐Analyses for Diagnostic Test Accuracy), this systematic review and meta‐analysis evaluated the accuracy of MRI in diagnosing DM tears [25]. A PROSPERO‐registered predefined protocol was used for this study (The registration number has been deleted to maintain manuscript blindness).

### Search strategy

Two independent reviewers (AM and AP) searched Embase, PubMed, Scopus, Web of Science and Google Scholar databases using keywords; ‘discoid’ AND (‘menisci’ OR ‘meniscus’) AND (‘tear’ OR ‘tearing’ OR ‘torn’ OR ‘injury’ OR ‘injuries’ OR ‘injured’ OR ‘damage’ OR ‘damaging’ OR ‘damaged’ OR ‘laceration’ OR ‘flap’ OR ‘defect’ OR ‘Thin’ OR ‘Fissure’ OR ‘degenerated’ OR ‘degenerative’ OR ‘degeneration’ OR ‘lesion’) AND (“MRI’ OR ‘magnetic resonance’) from inception of databases to November 2023.

### Eligibility and study selection

Studies that investigated DM using MRI and arthroscopic findings were included. Original studies and case series with at least ten patients were included in the study. Additionally, included studies were required to provide sufficient data for analysis, such as the number of patients diagnosed with DM through arthroscopy and MRI. The exclusion criteria were: (1) case reports and case studies; (2) reviews (narrative or systematic or meta‐analyses); (3) cadaveric studies; (4) animal studies; (5) consensus statements and guidelines; (6) editorial notes, letters and conference abstracts; (7) case series with <10 patients and (8) studies with insufficient data. Two independent authors (AP and SA) reviewed every report.

### Quality assessment

To assess the risk of bias and the applicability of the included studies [46], two independent authors (AM and SA) used the Quality Assessment of Diagnostic Accuracy Studies‐2 (QUADAS‐2) tool. The level of evidence (LOE) of this study was evaluated by accepted guidelines, which, due to including original articles with LOE I and II, the LOE of this study is II [24, 48].

### Data extraction

A predefined data extraction sheet was used to extract relevant information, including the number of patients, age, gold standard, true positives, true negatives, false negatives, and false positives of MRI for diagnosing DM. Two authors (MZM and AP) were responsible for data extraction, with controversial cases referred to a third reviewer (AM) for clarification.

### Data synthesis and meta‐analysis

Analysis was conducted using Stata version 17 (Stata Corporation) and Meta‐DiSc 1.4 (Ramon y Cajal Hospital). Based on pooled diagnostic performance, diagnostic odds ratios (DORs), positive likelihood ratios (PLRs) and negative likelihood ratios (NLRs) were estimated with 95% confidence intervals (CIs). For heterogeneity, the Cochrane *Q* test with *p* < 0.05 or *I* ^2^ > 50% implies heterogeneity. As a result, the DerSimonian–Laird random‐effect models were used in the current study due to significant heterogeneity [13]. Publication bias was assessed using Begg's test and funnel plot. The articles with insufficient data to create the two‐by‐two contingency table were also excluded. A study by de Hond evaluated most of the labelling systems for the thresholds to classify accuracy, sensitivity and specificity. The accuracy of 50–60, 60–70, 70–80, 80–90 and 90–100 was labelled as Failed (random), Poor (weak or low), Moderate (fair or acceptable), Good, Excellent (very good or high), respectively [7].

## RESULTS

### Literature search and characteristics of the studies

**Figure 1:**
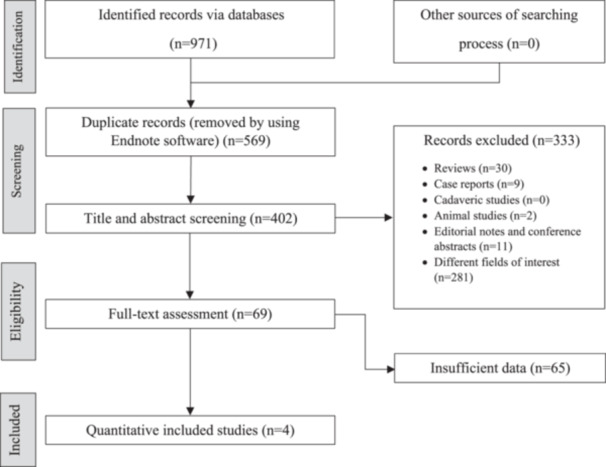
PRISMA flow chart for study selection.

**Figure 2:**
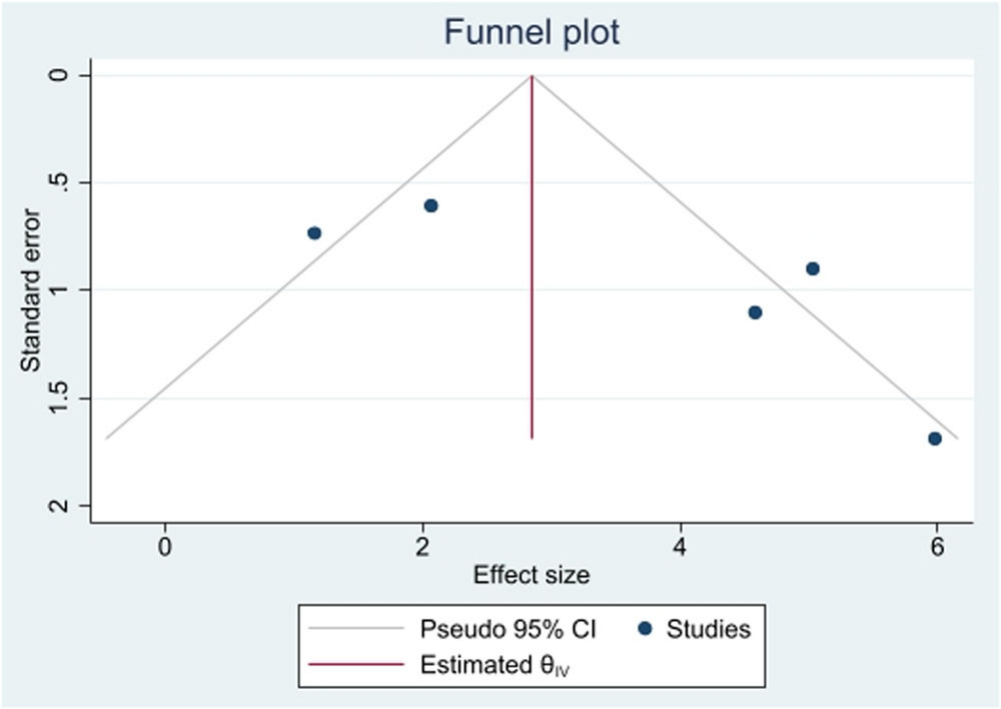
Funnel plot on the diagnostic accuracy of MRI for discoid meniscus tear. MRI, magnetic resonance imaging.

Figure 1 presents the flowchart of the current systematic review. A total of 971 articles were found in the initial search. In the next step, 569 articles were removed as duplicates by the Endnote software (version 20, Clarivate Analytics). Based on the title and abstract screening, 333 out of 402 reports were excluded. Subsequently, 65 studies were excluded during the full‐text review based on the exclusion criteria. Ultimately, four studies were included. In the Sun et al. study, two readers analysed two sets of MRI, so we mentioned them as two separate studies. Evaluation of publication bias was based on funnel plot and Begg's test (Figure 2). A summary of the study and patients' characteristics is presented in Table 1. An evaluation was conducted on four retrospective studies involving 305 patients. As a gold standard, arthroscopic findings were used in all studies. Teenagers and women were the major groups of patients included in the studies.

**Table 1:** Characteristics of the included studies.

Lead author	Year	Country	Study period	Study design	No. of cases	Age (years)	Mean age (years)	Male, %	Gold standard
C Yilgor [50]	2014	Turkey	1999–2009	Retrospective	52	5–59	26	46.1	Arthroscopy
WJ Yoo [52]	2012	South Korea	June 2003 to September 2010	Retrospective	73	4.3–17.6	10.9	39.72	Arthroscopy
XX Sun (a) [41]	2017	China	May 2009 to October 2015	Retrospective	73	8–16	13.8	46.5	Arthroscopy
XX Sun (b) [41]	2017	China	May 2009 to October 2015	Retrospective	73	8–16	13.8	46.5	Arthroscopy
BC Lau [21]	2018	USA	November 2011 to September 2016	Retrospective	34	8–16	8‐16	38.2	Arthroscopy

### Quality assessment and publication bias

QUADAS‐2 was used to assess the bias and applicability concerns for the included studies. The evaluated studies met at least four of the seven QUADAS‐2 criteria (Table 2). Evaluation of the included studies using the funnel plot showed no publication bias. Similarly, Begg's test revealed no significant publication bias (*p* = 0.46) (Figure 2).

**Table 2:** Quality Assessment of Diagnostic Accuracy Studies‐2 domains for the included studies.

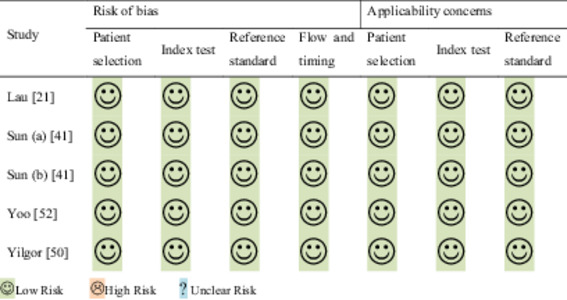

### Diagnostic performance of MRI for DM

**Figure 3:**
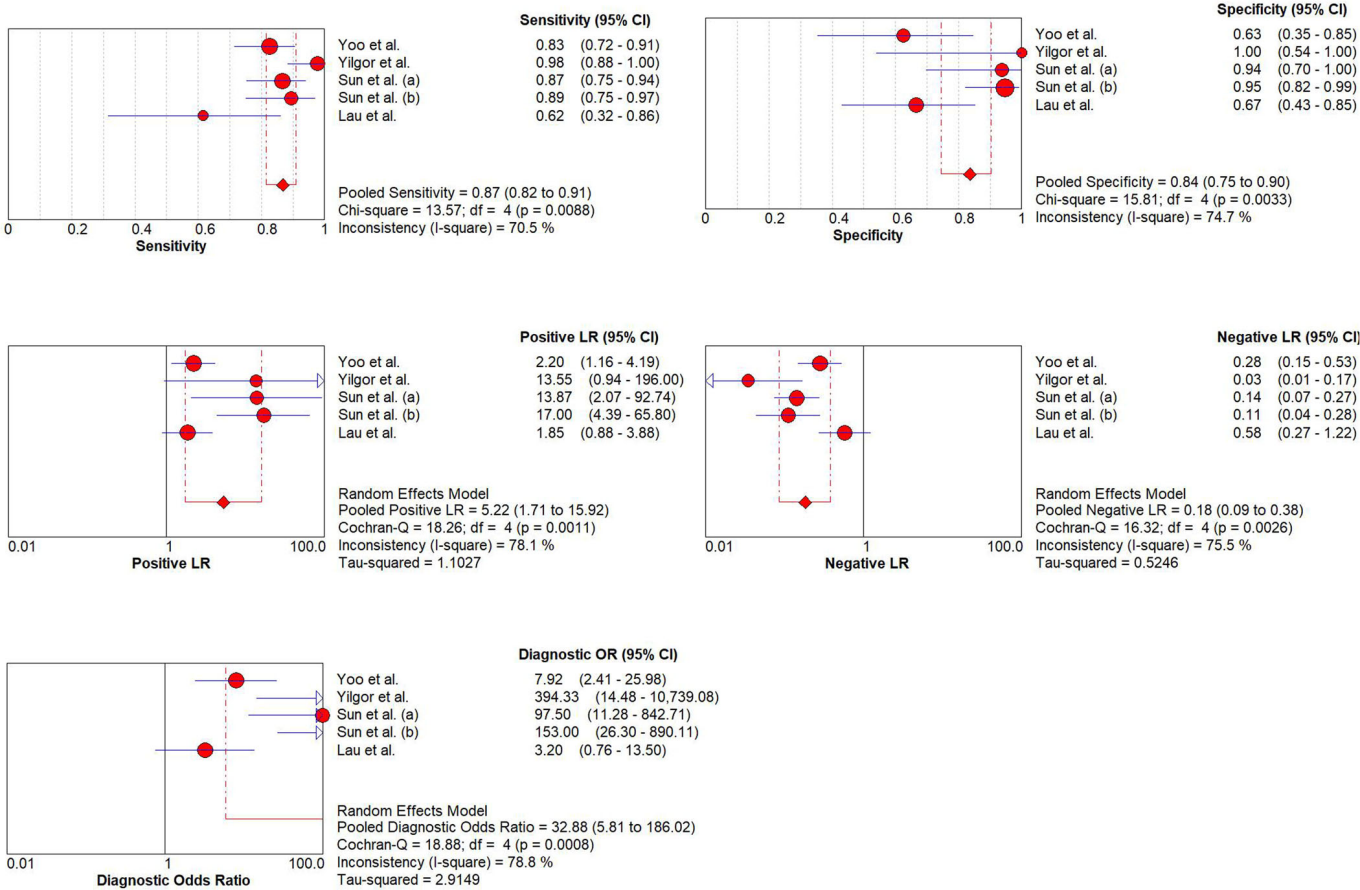
Forest plot of sensitivity, specificity, positive likelihood ratio (LR), negative LR and diagnostic odds ratio (DOR) of magnetic resonance imaging for discoid meniscus tear diagnosis and SROC curve are coupled. The points seen in each graph show the values mentioned above. 95% Cl for each included study represented by the horizontal line. The random effects model is indicated by a diamond shape in each graph. Information on pooled sensitivity, specificity, negative LR, positive LR, DOR, Chi‐square, Cochrane‐Q and inconsistency are shown at the bottom right of each graph. Corresponding heterogeneities (*I*²) with 95% Cl: *I*² = 100% × {(*Q* – *df*)/*Q*}, where *Q* is the Cochrane heterogeneity statistic and *df* indicates the degrees of freedom. CI, confidence interval.

**Figure 4:**
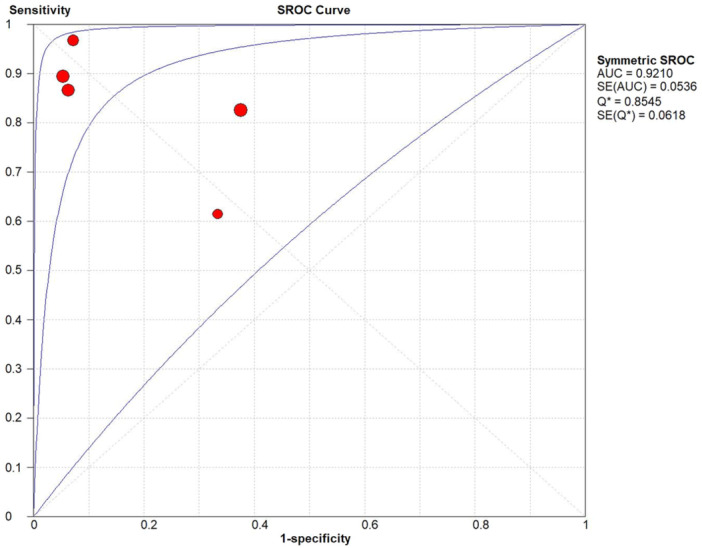
The summary receiver operating characteristic (SROC) plot was used to evaluate the diagnostic accuracy of MRI for ramp lesions, and the area under the curve (AUC) was 0.92. MRI, magnetic resonance imaging.

Five studies from four articles were reviewed with specificity and sensitivity ranging from 0.63 to 1.00 and 0.62 to 0.98, respectively. The pooled sensitivity, specificity, diagnostic OR, positive LR and negative LR were 0.87 (95% CI, 0.82–0.91), 0.84 (95% CI, 0.7–0.90), 32.88 (95% CI, 5.81–186.02), 5.22 (95% CI, 1.71–15.92) and 0.18 (95% CI, 0.09–0.38), respectively (Figure 3). The area under the hierarchical summary receiver operating characteristic curve was 0.92 (Figure 4).

The assessment of the heterogeneity of the diagnostic characteristics showed a significant heterogeneity for sensitivity (*I* ^2^ = 70.5% and *p* < 0.01), specificity (*I* ^2^ = 74.7% and *p* < 0.01), PLR (*I* ^2^ = 78.1% and *p* < 0.01), NLR (*I* ^2^ = 75.5% and *p* < 0.01) and DOR (*I* ^2^ = 78.8% and *p* < 0.01), respectively. The absence of a typical ‘shoulder arm shape’ of SROC and Spearman correlation coefficient between the log of specificity and sensitivity did not show any statistically significant threshold effect (*p* = 0.96).

## DISCUSSION

Based on this meta‐analysis study, MRI has a sensitivity of 87%, specificity of 84% and accuracy of 92% for diagnosing DM tears. DM is classified into three categories based on the Watanabe classification: complete DM, incomplete DM and menisci of the Wrisberg type (DM without normal posterior meniscotibial attachments) [44]. Reduction in collagen fibres and heterogeneous arrangement of these fibres within DM leads to DM tearing in trauma [42]. Many aetiological and pathophysiological factors are related to meniscal tears, such as sex, age, sports and degeneration [23, 37, 43]. Based on the modified O'Connor's classification system [4, 49], tear type could be categorized into one of the following groups: (1) a simple horizontal tear; (2) a combined horizontal tear, where the primary tear component is horizontal, accompanied by another tear component; (3) a longitudinal tear, which includes a peripheral tear; (4) a radial tear, involving an oblique and a flap tear; (5) a complex tear, which encompasses a degenerative tear, formed by combining two major components except for a horizontal tear, or a combination of three or more major tear components, including a horizontal tear and (6) a central tear.

The included studies evaluated MRI for two main changes: signal intensity change and morphological change. The classification of signal changes in the DLM was performed using a grading system: Grade 0 indicating no change, Grade 1 for dot‐like intrameniscal signal intensity, Grade 2 for linear or band‐like intrameniscal signal intensity and Grade 3 for linear or band‐like signal intensity extending to the superior or inferior meniscal surface. Additionally, a category termed ‘diffuse change” was defined as a diffuse increase in signal intensity in the DLM. This diffuse change was the most commonly observed signal change among the patients but could not be evaluated using the previous classification scheme. Furthermore, the morphologic changes (deformation or displacement) of menisci were classified based on the study by Ahn et al., with some modifications. In this classification, DM was considered to have undergone morphological changes when either (1) there was a greater than 70% difference in meniscal thickness between the anterior and posterior segments on sagittal images or between the medial and lateral segments on coronal images or (2) the meniscal segment crossed either the lateral tibial spine on coronal images or the tibial plateau margin on sagittal images. The morphologic changes of menisci were categorized as none, anterior, posterior, central, anterocentral or posterocentral [1].

In a study by Yoo et al., MRI is not considered a definitive tool in decision‐making regarding DLM tears but rather a complementary tool to careful physical examinations and arthroscopy. They found out that based on signal change‐based classification schemes, grade 3 changes are correlated with meniscus tears (accuracy of 96% and positive predictive value of 92%) [6]. In patients showing signal changes other than Grade 3, morphology change characterized in MRI did not accurately diagnose DM tears (sensitivity of 76% and negative predictive value of 44%). Preoperative MRI assessments relying on signal intensities do not reliably predict the occurrence of a DLM tear in children, except in cases where a DLM exhibits a Grade 3 signal change. However, meniscal deformation or displacement observed in preoperative MRI indicates an elevated likelihood of meniscal tears, even when the signal changes in the menisci are of grades other than 3 [52].

According to Park et al. [31], MRI is accurate for detecting DM but less helpful for DM tears. They showed that MRI was 100% sensitive in identifying DM in clinically suspicious patients, consistent with previous literature reports [2]. In contrast, MRI was only 50% specific and 75% sensitive in identifying torn DM, with a high incidence of false‐positive and false‐negative findings [11]. In the adolescent population, changes in vascularity within the meniscus can explain the false‐positive rate presented in their study. Meniscal tears are frequently misinterpreted due to these vascularity changes in the meniscus [5, 6]. Ryu et al. also found that MRI was unreliable for identifying DM tears. They reported that the PPV was 92% and 57% for DM and DM tears, respectively, where the torn segments were displaced in 51 patients (72%) [34]. However, diagnosis of DLM instability on MRI is challenging due to the dynamic characteristics of the DLM, including instances of peripheral detachment and incomplete DLM signs mimicking a normal meniscus [17], which may be a possible explanation for the reported false negative results. Lau et al. evaluated the accuracy of MRI in predicting DM injuries. The sensitivity and specificity of MRI in their study were 60% and 66.7%, respectively. A DLM with abnormal anatomy may explain this difficulty in identification by MRI. Factors such as insurance confirmation for requesting an MRI study, MRI availability and decision‐making result in a delay between an MRI study and surgery, which may lead to a discrepancy between MRI findings and surgery. A delay in MR imaging to surgery did not significantly differ between groups, but this delay could lead to a secondary injury or development or worsening of articular damage during this time [21].

DM leads to some changes and causes some indirect signs of DLM, including widening of the lateral joint space, squaring of the lateral femoral condyle, cupping of the lateral tibial plateau, lateral tibial eminence hypoplasia, elevation of the fibular head and condylar cutoff sign [9, 10, 19, 30, 32].

Some studies support MRI as a high‐accuracy modality for detecting DM tears. The number of studies that agreed with our study was higher than those that disagreed. In a study conducted by Jung et al [16]., sensitivity, specificity and positive predictive values of 95% (95% CI, 85%–99%), 100% (54–100) and 100% (93–100) were reported in children, respectively, while reporting 97% (97%–99%), 100% (31–100), and 100% (96–100) for the same indices in adults, respectively. Their results demonstrated a more significant MRI accuracy than our pooled results. Another study by Jung et al. [50]. found that MRI was 100% sensitive and 97.8% specific for diagnosing DM tears. The NPV and PPV of MRI for predicting a tear were 85% and 100%, respectively. In a different study, researchers found that MRI had a 75% sensitivity and 50% specificity for identifying DM tears [11].

Besides diagnostic accuracy, the reliability of a modality plays an important role in clinical implications. Niu et al. evaluated the intraobserver and interobserver reliability of using MRI as a diagnostic modality for 45 knees with DM injuries. They showed inter‐ and intraobserver reliability in assessing most characteristics of DM injuries with MRI ranging from substantial to moderate. Among the different types of DM Injuries, DM tears have fair interobserver and substantial intraobserver reliability [29].

The analysis revealed considerable heterogeneity in the results section. Numerous factors contribute to this finding [45]. Initially, the demographic characteristics of the participants in the research varied significantly with respect to both age and gender, covering a wide span of ages from 4 to 59 years. Second, various MRI signs were evaluated in the studies included, underscoring the requirement for future investigations to examine each sign independently. Furthermore, the studies incorporated varying levels of quality, such as sample size differences, highlighting the necessity for additional research with larger sample sizes in this field.

There are limitations associated with this study. Although most studies focused on paediatric and teenage patient populations, one study covered adult patients, which might present bias. In addition, the study involved only a small number of patients and most of them were women. Therefore, if future studies are conducted on a larger and more representative sample of patients, the results will be more reliable.

## CONCLUSION

MRI has been found to have excellent sensitivity and specificity as a diagnostic tool for DM tears based on a meta‐analysis of contemporary studies. Even though MRI has lower accuracy than arthroscopy, it can be used in high‐risk patients instead of arthroscopy due to its advantages.

## AUTHOR CONTRIBUTIONS

Alireza Mirahmadi, Shayan Amiri, Seyed Morteza Kazemi and Kaveh Momenzadeh contributed to the study design, data collection and drafting of the study. Alireza Mirahmadi, Shayan Amiri and Mana Zaker Moshfegh designed the search strategy. Seyedeh Pardis Hashemi Abatari and Ava Parvandi did the quality assessment. Mehrdad Farrokhi did the statistical analysis. Alireza Mirahmadi, Shayan Amiri, Seyed Morteza Kazemi, Kaveh Momenzadeh, Jim S. Wu and Ara Nazarian conducted the final revision. All authors critically reviewed the content and approved the final version for publication.

## CONFLICT OF INTEREST STATEMENT

The authors declare no conflict of interest.

## ETHICS STATEMENT

The ethics statement is not available. There is no information (names, initials, hospital identification numbers or photographs) in the submitted manuscript that can be used to identify patients.

## Data Availability

Data will be made available on request.
